# Suspension

**Published:** 1861-10

**Authors:** 


### Suspension.

With this issue of the Journal we are forced to suspend its publication *temporarily.* This we deeply regret from many considerations. In the first place it is exceedingly unpleasant to falter in the prosecution of an enterprise, under any circumstances; and though the public mind, among the medical, as well as all other professions, is absorbed with our national difficulties, and the energies of all directed toward the maintainence of our independence, it is nevertheless painful, even for a time, to give up the means of communication with the profession, so long enjoyed. Since the existence of the war, the interest in the work, on the part of the Editor and patrons, has to some extent been lost; and whatever of shortcoming in its publication, and quantity and quality of its matter for its pages, has been observed by its readers, we hope will not be attributed to wanton negligence on the part of the Editor, but to the additional duties we felt it our duty to perform in the army, and which we do not feel at liberty to decline now, and may not for some time to come.

To the kind friends who, in the four or five months' absence of the Editor have assumed the editorial labor, our warmest gratitude is due; and while their pressing professional engagements would not allow the requisite amount of time for its management, it is believed that every patron of the work will feel satisfied that, under the circumstances, the Journal has lost nothing by the change.

Further tax, however upon the time of those who would willfully devote every spare moment to the interest of the Journal, we cannot get our consent to ask. The issue therefore must be suspended till our Governmental troubles abate, or until our services are no longer required in the army. The Journal is not dead, but sleeps. The time will come, ere long, and we may hope in a few months—when such enterprises will awake from their war-slumber, and science again march forward in her majesty.

To those patrons who have paid for the seventh volume in advance, the amount for the balance of the volume will be forwarded, lest the time of suspension may be longer than is desirable for advance payment.

From those in arrears for the Journal we hope to receive the amount for which we have toiled, and which would now greatly relieve us.

